# Novel homozygous mutation in the human *RAX* homeobox gene in a patient with bilateral anophthalmia and severe endocrine dysfunction – a case report and literature review

**DOI:** 10.1515/crpm-2024-0018

**Published:** 2024-09-12

**Authors:** Yasmin H.A. El-Nahry, Victor Bardinet, Christoph Bührer, Wolfgang Henrich

**Affiliations:** Department of Obstetrics, Charité – Universitätsmedizin Berlin, Berlin, Germany; Department of Neonatalogy, Charité – Universitätsmedizin Berlin, Berlin, Germany

**Keywords:** anophthalmia, congenital hypopituitarism, endocrine dysfunction, neonatale diabetes insipidus, *RAX* gene

## Abstract

**Objectives:**

Childhood visual impairment due to congenital malformation leads to severe handicaps and lifelong consequences for the affected child. Congenital anophthalmia remains a rare condition marked by a child born with an empty eye socket. The embryonic plant of the eye occurs approximately on day 22 of intrauterine development and ends within the first trimester of pregnancy. Mutations in the *RAX* gene located on chromosome 18 (# 601881) cause a spectrum of head malformations, ranging from isolated microphthalmia/anophthalmia with cleft lip and palate to complex brain malformations.

**Case presentation:**

Here, we present a child’s case diagnosed with bilateral anophthalmia at 33 weeks of gestation. The newborn was delivered vaginally with a *RAX*-gene-linked syndrome. Besides craniofacial malformations (bilateral anophthalmia, craniofacial hypoplasia, bilateral cleft lip), the female child had severe endocrine dysfunction (congenital hypopituitarism and diabetes insipidus) postnatal that required specialised monitoring and clinical management. Our case study reports a novel homozygous autosomal recessive non-sense mutation (c.106G>T; p.Glu36Ter) of the *RAX* gene. This is the first description of this pathogenic gene variant in the literature.

**Conclusions:**

Early and precise sonography is crucial in detecting these conditions on time to prepare postpartum care and avoid delays in optimal clinical treatment for the affected child. This case report aims to raise the scientific community’s awareness about this rare genetic syndrome, showing an individualised two-year follow-up program that could help guide physicians and future parents of affected children.

## Introduction

Congenital malformations of the ocular system account for a significant proportion of childhood visual impairment worldwide, with anophthalmia being a rare but severe ocular abnormality. Ocular abnormalities profoundly impair the affected children and their families’ physical, emotional, and social development, causing a certain economic cost for society. The estimated birth prevalence of anophthalmia and microphthalmia ranges from 3 to 14 per 100.000 births. The causes of anophthalmia among most infants during pregnancy are unknown. In a majority of severe bilateral cases, a single gene disorder or chromosomal disorders are diagnosed during pregnancy or shortly after birth. According to Mathers et al., Homeobox-containing genes are crucial for the formation of both vertebrate and invertebrate eyes [1]. While SOX2 (sex-determining region Y-box 2) is the most common gene associated with anophthalmia [2], 3, mutations in OTX2 (orthodenticle homeobox 2) or PAX6 (paired box 6) genes can also cause this ocular abnormality [3], [4], [5]. It has been shown that Homeobox-containing genes, such as *RAX*, play a vital role in the formation of both vertebrate and invertebrate eyes. The misexpression of *RAX* can significantly affect eye development [1]. However, pathogenic variants in *RAX* are described less frequently in literature, but cause a broad spectrum of craniofacial deformation to complex brain malformations.

## Case presentation

**Figure 1:**
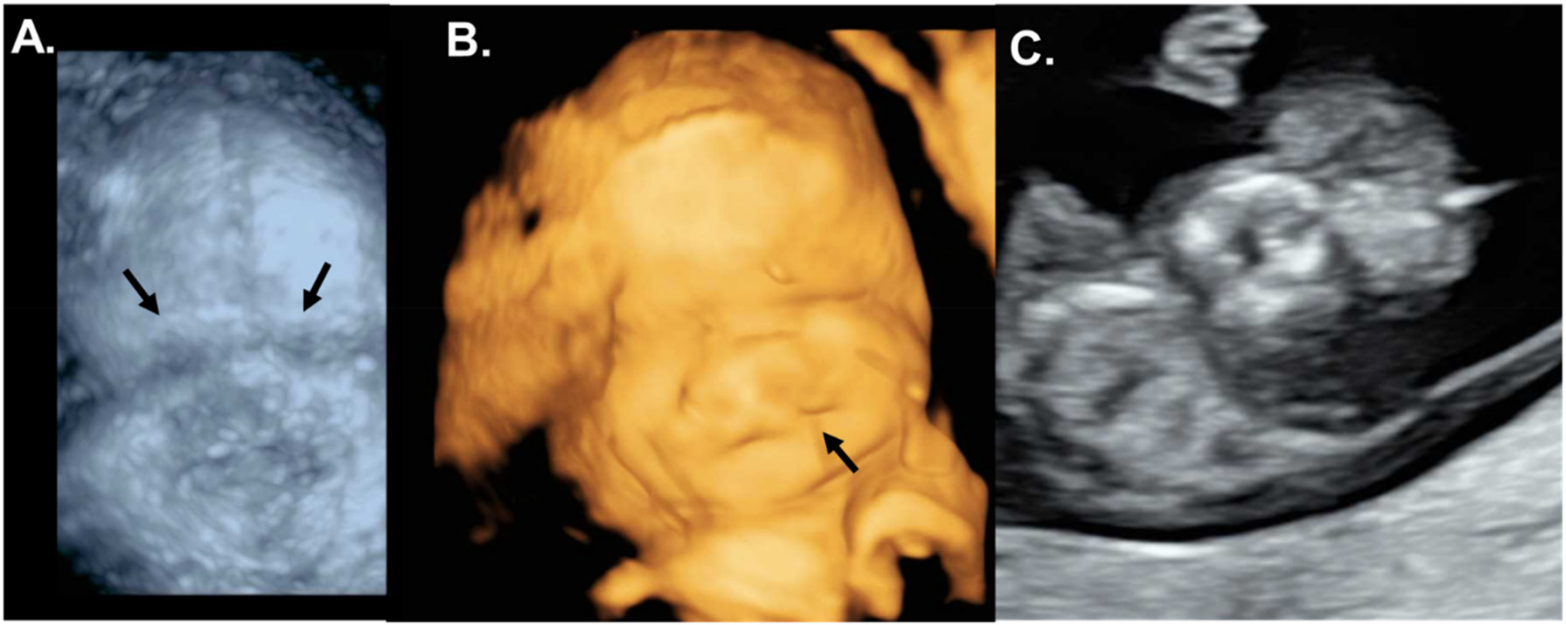
Antenatal ultrasounds showing the facial characteristics at different stages of pregnancy. (A) 3D static ultrasound (skeletal mode) of the skull in frontal view, with the missing representation of the orbits (marked with arrows) at 32 + 4 weeks of gestation. (B) 3D surface view of the face showing conspicuously low-lying eyelids and a cleft lip (marked with an arrow) at 32 + 4 weeks of gestation. (C) 2D frontal view of the fetal facial skull during first-trimester screening, with missing orbits at 11 + 2 weeks of gestation in the subsequent pregnancy.

**Figure 2:**
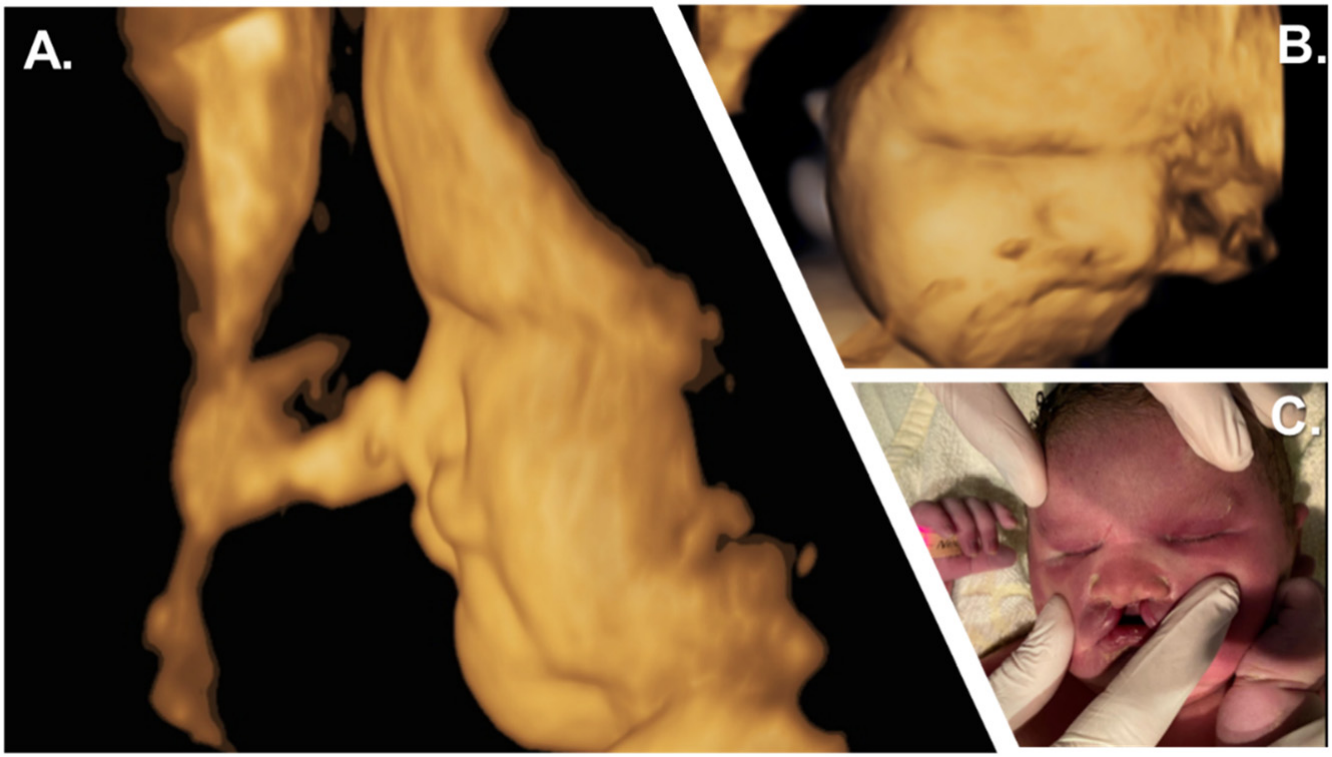
Prenatal and postnatal facial characteristics. (A, B) 3D surface sagittal view of the child’s face at 39 weeks of gestation showing craniofacial hypoplasia with a flat nose. (C) Initial examination showed the neonate’s fused eyelids, bilateral cleft lip and jaw with a suggestive philtrum, complete absence of premaxilla, and total vomer aplasia.

A female child was born post-term, after 43 + 4 weeks of gestation, from consanguineous parents. The mother had a normal antenatal course until 32 + 4 weeks of gestation, when a prenatal sonogram detected bilateral anophthalmia with a cleft lip and palate and craniofacial midline hypoplasia (Figure 1, Figure 2A and B).

**Figure 3:**
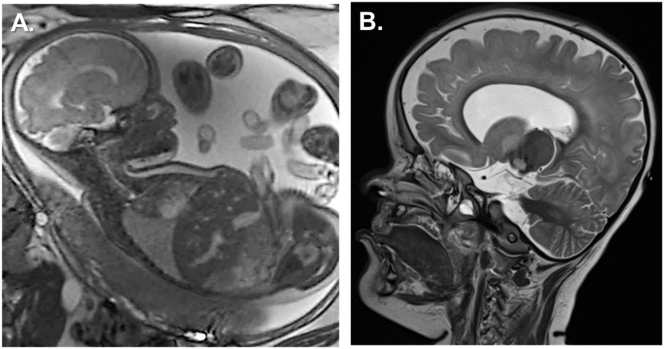
Fetal and cerebral magnetic resonance imaging (MRI). (A) Fetal MRI at 32 weeks showed: flat orbital roof, suspected hypoplastic orbital conus, hypoplastic bulb on both sides, shortened nasal septum, flat nose and bilateral cleft lip. Anophthalmia and microphthalmia were undifferentiated. (B) T2-weighted MRI of the central nervous system at age two showed: brain parenchymal hypoplasia, extensive diffusion restriction to the mesencephalon, bilateral anophthalmia with aplasia of the anterior visual pathway, non-developed pituitary gland causing panhypopituitarism.

**Figure 4:**
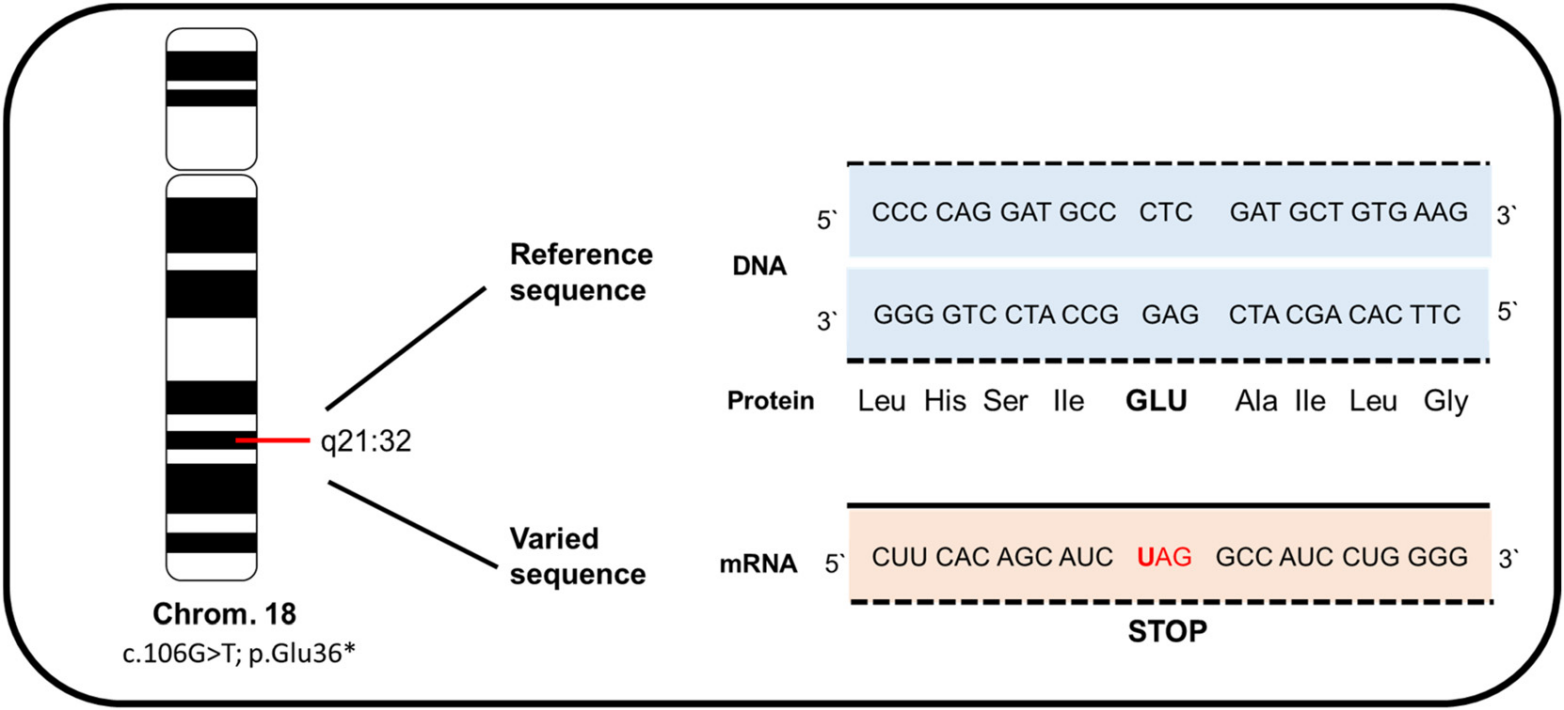
Illustration of the homozygous variant c.106G>T; p.Glu36* in the *RAX* gene located on chromosome 18 from our patient. This mutation leads to the formation of a stop codon, causing a nonsense-mediated mRNA decay and resulting in a loss of protein function.

The diagnosis of anophthalmia was confirmed by fetal magnetic resonance imaging (MRI) (Figure 3A). Clinical exome sequencing (CES) was performed after amniocentesis, revealing a novel homozygous autosomal recessive mutation in chromosome 18. According to genetic analysis, this *RAX* mutation (c.106G>T; p.Glu36Ter) within the *RAX* gene formed a stop codon, causing a nonsense-mediated mRNA decay and resulting in a loss of function of the protein (Figure 4).

Although the expectant parents discussed the possibility of pregnancy termination, they ultimately decided to continue by mutual agreement. The possibility of induction was considered at 40 + 6 weeks of gestation if labor had not yet commenced. However, the mother did not return to the physician until 43 + 4 weeks of gestation. After receiving prostaglandin induction, a post-term baby girl weighing 4,560 g (>97th percentile) was delivered vaginally, with a birth length of 58 cm (>97th percentile) and a head circumference of 38 cm (>97th percentile). The amniotic fluid was meconium-stained, and the umbilical artery pH was 7.37. The child’s Apgar score was 8/9/10 after 1, 5, and 10 min of life.

After delivery, postpartum sonography and complete ophthalmologic examination confirmed the absence of ocular tissue in the orbits and a complete fusion of the eyelids. The front part of the child’s brain, including the eyeballs and outgoing optic nerves, was not visualized. Clinical examination revealed cleft lips on both sides, midline craniofacial hypoplasia (Figure 2C), and hepatosplenomegaly. Due to several episodes of desaturation, the newborn was admitted to the neonatal intensive care unit for respiratory distress but recovered within a few hours. Repeated cerebral ultrasound failed to visualize a pituitary gland at two days of age. An additional laboratory assessment for endocrinological dysfunction revealed congenital panhypopituitarism. At nine days of age, the newborn presented with severe hypernatremia and polyuria, leading to the diagnosis of central diabetes insipidus. Echocardiography revealed temporary pulmonary hypertension with tricuspid valve insufficiency, right ventricular hypertrophy, and right ventricular dysfunction. Additional abdominal ultrasonography showed hepatosplenomegaly, a disproportionately large uterus, and absent adrenal glands. Further examinations were performed, including chest and abdominal radiography, whole-body sonography, brainstem evoked response audiometry and newborn screening – all revealed normal findings. During hospitalisation, the infant developed two episodes of neonatal infections and icterus praecox, which were treated with antibiotics and phototherapy, respectively. She also presented several episodes of hyperthermia without evidence of either infection or inflammation, which were interpreted as hypothalamic temperature instability retrospectively. The newborn was discharged home at 43 days of age with outpatient ambulatory care.

## Follow-up

After discharge, the family was enrolled in a follow-up program at our paediatric outpatient clinic. Within six months, the girl showed severe psychomotor delay, hypotonia, and abnormal leg movements. She was diagnosed with congenital panhypopituitarism and started lifelong supplementation with hydrocortisone (4 mg/day) and l-thyroxine (50 µg/day). The central diabetes insipidus was treated with intranasal desmopressin (8 μg/d). Repeatedly performed EEG did not find any seizure-like electrical activity. The child was hospitalised and released home several times with metabolic derailment, endocrine dysregulation, and episodes of fever due to viral respiratory infections. At the age of 9 months, an examination of the cleft lip under anaesthesia confirmed partial agenesis of the maxillary bone. The first correction of the cleft lip took place at 12 months, with lip plastic and gingivo periosto plastic surgery, followed by a final correction at 24 months. The child experienced postoperative complications, including infection and severe decompensation of diabetes insipidus, leading to hypovolemia. In the course, the infant developed severe hypernatremia (up to 168 mmol/L) and rhabdomyolysis with peak concentrations of myoglobin reaching up to 1722 μg/L (Ref. 25–58 μg/L), lactate dehydrogenase level of 1394 U/L (Ref. 202–344 U/L), and creatine kinase level of 8986 U/L (Ref. <167 U/L). A later performed cerebral MRI at age two revealed a diffuse pathological signal with atrophy and hypoplasia in most parts of the brain parenchyma and aplasia of the pituitary (Figure 3B).

Due to the acute clinical deterioration of the child and the poor prognosis, parents and physicians decided to redirect care at the age of two years and 4 months. The infant was transferred to a children’s hospice, where she lived until she turned three years old. Her mother got pregnant again. An early ultrasound diagnostic revealed the same ocular abnormality in the first pregnancy, followed by the parents’ decision to end the pregnancy without performing a genetic testing of the fetus (Figure 1C).

## Discussion

During the development of the vertebrate eye, a series of intricate steps must occur to form the visual system, including the specification of the anterior neural plate, the evagination of the optic vesicles from the ventral forebrain, and the cellular differentiation of both the lens and retina. Mathers et al. found that Homeobox-containing genes, particularly *RAX*, play a critical role in forming vertebrate and invertebrate eyes. The misexpression of the Retinal homeobox protein Rx, also known as retina and anterior neural fold homeobox *RAX* that is located on chromosome 18 (OMIM No. #601881), can have profound effects on eye development and morphology, as this gene is responsible for the proper differentiation of the lens and retina [1]. There are a few studies conducted by Voronina et al., Lequeux et al., Abouzeid et al., and Chassaing et al. [6], [7], [8], [9], [10] that provide valuable insights into the various *RAX* gene mutations showing patients’ cases with congenital eye malformations. Voronina et al. screened 75 individuals with clinical anophthalmia and microphthalmia for mutations in the *RAX* gene. They found that a 12-year-old autistic boy with clinical anophthalmia of the right orbit and microphthalmia with sclerocornea of the left eye had compound heterozygosity for a truncated allele (Q147X; 601881.0001) and R192Q mutation (601881.0002), both within the homeodomain of the *RAX* gene [6]. Lequeux et al. also identified compound heterozygosity for a deletion (601881.0003) and a nonsense mutation (601881.0004) in the *RAX* gene in a 2-year-old Algerian girl with bilateral clinical anophthalmia [7]. In two unrelated Egyptian families, Abouzeid et al. found three cases where individuals had bilateral anophthalmia with associated severe brain anomalies. In all three cases, homozygosity for the same splice site mutation (601881.0005) in the *RAX* gene was identified [8]. In a study by Chassaing et al., four patients with biallelic mutations (611038.0006) in the *RAX* gene were identified from a cohort of 150 patients with isolated or syndromic microphthalmia or anophthalmia [9]. Brachet et al. recently reported that the loss of expression of the *RAX* gene leads to endocrinological imbalances such as hypothyroidism, hypopituitarism, and diabetes insipidus, as well as bilateral lip and cleft palate. They identified a 1-bp deletion (611038.0007) in the *RAX* gene as well as a missense mutation of unknown significance in *RAX* in a 10-month-old boy with bilateral anophthalmia, cleft lip and palate, and hypoplasia of the anterior pituitary gland [10]. Additionally, Brachet was able to show using an animal model with knockout mice that experimentally generated *RAX*-null mice showed similar abnormalities in their newborn pups, such as a loss of ventral forebrain structures and the pituitary, basosphenoid bone, and palate.

Although congenital hypopituitarism (CH) can be associated with various eye or craniofacial anomalies [5], the co-occurrence of CH, anophthalmia, cleft palate, and diabetes insipidus has rarely been described. There is a narrow window of opportunity to treat a visually impaired infant. If not detected and managed promptly, these congenital malformations lead to severe developmental consequences. Brachet et al. reported that the loss of expression of the *RAX* gene can lead to endocrinological imbalances such as hypothyroidism, hypopituitarism, and diabetes insipidus, but also bilateral lip and cleft palate [10]. In our presented clinical case, we describe a rare patient’s phenotype with a new pathogenic variant in the *RAX* gene leading to the co-occurrence of anophthalmia with orofacial cleft and extraocular endocrine dysfunction. Our patient’s condition was diagnosed at a second glance at 32 + 4 weeks of gestation using two-dimensional (2D) ultrasonography during antenatal ultrasonography after referral to our hospital. To date, only one comparable case with a homozygous mutation of the *RAX* gene has been published that influenced our established management of clinical practice [10]. The evaluation of the literature indicates that some diagnosed children with anophthalmia presented a simultaneous endocrine disorder that was neither diagnosed nor treated at a time. According to Abouzeid et al., 2 out of 5 patients homozygous for the c.543 + 3A>G splice *RAX* mutation had died of dehydration [8]. Another homozygous child showed persistent polyuria-polydipsia symptoms, suggesting neonatal diabetes insipidus retrospectively. This patient’s description includes a detailed follow-up of a child with these abnormalities and aims to help guide physicians and future parents of affected children.

In conclusion, ocular malformations should be detected in early fetal screenings before birth. Anophthalmia diagnosis can be made simply by two-dimensional (2D) ultrasonography or by adding a third dimension (3D) if necessary, e.g. when the fetal head position is unfavourable. A prenatal suspicion of fetal anophthalmia requires precise genetic clarification using clinical exome sequencing (CES). Postnatal endocrinological diagnostics are obligatory after the detection of *RAX* mutation in neonates or children, as congenital anophthalmia has a high mortality due to severe extraocular endocrine dysfunction. Best Care provides a multidisciplinary team, including neonatologists, ophthalmologists and endocrinologists, for medical follow-up of affected children.

## Conclusions

The case highlights the importance of prenatal screening and genetic testing in identifying complex medical conditions early, allowing for appropriate management and treatment.

## Learning points

– Ocular malformations should be detected in early fetal screenings before birth.
– Anophthalmia diagnosis can be made simply by two-dimensional (2D) ultrasonography or by adding a third dimension (3D) if necessary, e.g. when the fetal head position is unfavourable.
– Prenatal suspicion of fetal anophthalmia requires precise genetic clarification using clinical exome sequencing (CES).
– In case of a *RAX* mutation, postnatal endocrinological diagnostics are obligatory.
– Best Care provides a multidisciplinary team, including neonatologists, ophthalmologists, and endocrinologists, following the children’s development.
